# Cost-effectiveness of camrelizumab plus chemotherapy vs. chemotherapy in the first-line treatment of non-squamous NSCLC: Evidence from China

**DOI:** 10.3389/fmed.2023.1122731

**Published:** 2023-02-14

**Authors:** Hongbin Dai, Wenyue Wang, Xin Fan, Yongfa Chen

**Affiliations:** School of International Pharmaceutical Business, China Pharmaceutical University, Nanjing, China

**Keywords:** non-small cell lung cancer, cost-effectiveness, camrelizumab, partitioned survival analysis, China

## Abstract

**Objective:**

We aimed to evaluate the cost-effectiveness of camrelizumab plus chemotherapy compared with chemotherapy alone as the first-line treatment for patients with metastatic or advanced non-squamous non-small cell lung cancer (NSCLC) without targetable epidermal growth factor receptor or anaplastic lymphoma kinase genetic aberrations in patients in China.

**Methods:**

A partitioned survival model was constructed to estimate the cost-effectiveness of camrelizumab plus chemotherapy vs. chemotherapy in the first-line treatment of non-squamous NSCLC from a Chinese healthcare perspective. Survival analysis was performed to calculate the proportion of patients in each state using data from trial NCT03134872. The cost of drugs was obtained from Menet, and the cost of disease management was obtained from local hospitals. Health state data were obtained from published literature. Both deterministic sensitivity analyses (DSA) and probabilistic sensitivity analysis (PSA) were adopted to verify the robustness of the results.

**Results:**

Compared with chemotherapy alone, camrelizumab plus chemotherapy provided 0.41 incremental quality-adjusted life years (QALYs) at an incremental cost of $10,482.12. Therefore, the incremental cost-effectiveness ratio of camrelizumab plus chemotherapy was $25,375.96/QALY from the Chinese healthcare perspective, much lower than three times the GDP per capita of China in 2021 ($35,936.09) as the willingness-to-pay threshold. The DSA indicated that the incremental cost-effectiveness ratio was most sensitive to the utility value of progression-free survival, followed by the cost of camrelizumab. The PSA illustrated that camrelizumab had 80% probability of being cost-effective at the threshold of $35,936.09 per QALY gained.

**Conclusion:**

The results suggest that camrelizumab plus chemotherapy is a cost-effective choice in the first-line treatment for patients with non-squamous NSCLC in China. Although this study has limitations such as short time of use of camrelizumab, no adjustment of Kaplan–Meier curves and the median overall survival that has not been reached, the difference in results caused by these factors is relatively small.

## 1. Introduction

Lung cancer is the leading cause of cancer-related deaths in China and worldwide. According to the *2020 Global Cancer Report* issued by the International Agency for Research on Cancer under the World Health Organization, the number of new cases of lung cancer worldwide has reached 2.2 million, second only to breast cancer; with 1.8 million deaths, its fatality far exceeds other cancers and ranks first. The number of new cases and deaths from lung cancer in China increased to 820,000 and 710,000, accounting for 17.9 and 23.8%, respectively. Lung cancer is one of the most common malignant tumors that seriously affects human health (1).

Lung cancer is divided into small cell lung cancer (SCLC) and non-small cell lung cancer (NSCLC), of which NSCLC accounts for up to 80–85% of the cases (2). However, owing to the insidious onset and lack of early diagnosis, 50% of NSCLC patients present with locally advanced or metastatic disease at the time of diagnosis (3). With the rapid development of immunotherapy, the first-line treatment for lung cancer has gradually changed from chemotherapy alone to PD-1/PD-L1 inhibitor plus chemotherapy. Studies have shown that blocking the combination of PD-1 and PD-L1 can ensure immune activation of T lymphocytes and kill tumor cells (4).

On June 19, 2020, camrelizumab was approved by the National Medical Products Administration for the first-line treatment of metastatic or advanced non-squamous NSCLC without targetable epidermal growth factor receptor (EGFR) or anaplastic lymphoma kinase (ALK) genetic aberrations, based on trial NCT03134872. NCT03134872 is a randomized, open-label, multicenter, phase III trial designed to evaluate the safety and efficacy of camrelizumab plus chemotherapy (carboplatin+pemetrexed) compared with chemotherapy alone (carboplatin+pemetrexed) in patients with advanced/metastatic non-squamous NSCLC without EGFR or ALK genetic aberrations (5). The results of the trial show that camrelizumab plus chemotherapy has significant clinical benefits: the median progression-free survival increased from 8.30 months to 11.30 months; the objective response rate (ORR) increased from 39.1 to 60%; median overall survival (OS) increased from 20.9 months to >21 months; and the safety is acceptable (5).

For camrelizumab, there are several studies assessing the cost-effectiveness by partitional survival model, and Markov model, respectively (6–11). However, there are two areas that need to be explored: First, the regimen of second-line treatment was not mentioned in the Camel trial, so second-line treatment is based on assumptions, which has a significant impact on the outcome. Second, the utility in some studies were from western countries, which also have a large impact on the results.

In this study, using NCT03134872 data, the partitioned survival model was designed to evaluate the cost-effectiveness of camrelizumab plus chemotherapy (carboplatin + pemetrexed) compared with chemotherapy alone in patients overall within the currently approved indication and from the perspective of the Chinese healthcare system, and the subsequent therapy of our study was also based on the subsequent 2-line therapy of Camel trial (NCT03134872) while other study's second-line treatment is based on assumptions. Also, the other purpose of this study is to compare the results with similar articles and explaining the reasons for the large differences.

## 2. Methods

### 2.1. Target population

The target population of the model was based on the NCT03134872 trial population. This trial included 412 lung cancer patients from 52 hospitals in China, all aged 18–70 years. The inclusion criteria were as follows: histologically or cytologically confirmed stage IIIB–IV non-squamous NSCLC; no previous systemic chemotherapy; no EGFR or ALK alteration; Eastern Cooperative Oncology Group performance status score of 0–1; had at least one measurable lesion per Response Evaluation Criteria in Solid Tumors (version 1.1); had no untreated brain metastasis; and had a life expectancy of at least 3 months (5).

### 2.2. Study perspective

This study will examine the cost-effectiveness of camrelizumab plus chemotherapy vs. chemotherapy alone from the perspective of the Chinese healthcare system.

### 2.3. Comparators

The trial was divided into a “camrelizumab plus chemotherapy” group and a “chemotherapy alone” group. A total of 412 patients with non-squamous NSCLC were randomly assigned.

- Camrelizumab plus chemotherapy group: 205 patients.

Camrelizumab 200 mg once every 3 weeks.
Pemetrexed 500 mg/m^2^ once every 3 weeks.
Carboplatin area under curve, 5 mg/mL per min once every 3 weeks.
followed by Maintenance camrelizumab and pemetrexed.

- Chemotherapy alone group: 207 patients.

Pemetrexed 500 mg/m^2^ once every 3 weeks.
Carboplatin area under curve, 5 mg/mL per min once every 3-weeks.
followed by Maintenance pemetrexed.

### 2.4. Time horizon

In the NCT03134872 trial, NSCLC patients received a drug injection every three weeks, so this study set the cycle period to 3 weeks, and according to the model validation results, after 104 cycles, 99% of the patients were already in the death state, therefore the number of cycles is set to 104 cycles.

### 2.5. Discount rate and willingness-to-pay threshold

According to the *China Guidelines for Pharmacoeconomics Evaluations* (2020), a discount rate of 5% per year was used for cost and utility, and 0–8% was used for deterministic sensitivity analyses (12). Three times the GDP per capita of China in 2021 ($35,936.09) was used as the willingness-to-pay (WTP) threshold (13).

### 2.6. Outcomes

The main outcomes of the model were the total cost in both treatment arms, quality-adjusted life year (QALY), and incremental cost-effectiveness ratio (ICER).

### 2.7. Cost and utility

#### 2.7.1. Cost data

Only direct medical costs were included in this study, and the prices of the above-mentioned medicines were sourced from MENET (14). In China, camrelizumab is available in single-use vials of 200 mg and injected at a fixed dose of 200 mg every 3 weeks. The list price of camrelizumab after accounting for medical insurance is $433.14, compared with $2,928.99 before. Pemetrexed is available in single-use vials of 500 mg, which cost $404.71 based on the standard dosage and price of pemetrexed using the average body surface area (mean = 1.72 m^2^) of the Chinese population (15). The cost per dose for pemetrexed was estimated to be $696.10(14). The price of carboplatin is $4.49 for 50 mg, and the total cost every 3 weeks is $67.34, when the area under curve is 5. It is assumed that no vial waste is associated with camrelizumab, pemetrexed, and carboplatin.

In addition to cost data of above medicines, there is also some disease management data which were collected from local hospitals. The costs of outpatient fees and blood tests were $3.70 and $7.40, respectively, once every 3 weeks. Both puncture and PD-L1 testing were performed once before starting treatment, and the prices were $443.79 and $295.86, respectively. The price of tumor imaging assessments was $73.96, which was done every 6 weeks for the first 54 weeks and every 12 weeks thereafter.

Based on the above costs, the cost input was finally including eight input parameters: one-time cost–camrelizumab, cost in progression-free survival-camrelizumab, cost in maintenance therapy–camrelizumab, cost in progressive disease-camrelizumab, one-time cost–chemotherapy, cost of progression-free survival-chemotherapy, cost of maintenance therapy-chemotherapy, and cost of progressive disease–chemotherapy. In 0–6 cycles (0–4.2 months), patients in the progression-free survival state used cost in progression-free survival, and if the disease progressed, the subsequent therapies began, and the cost changed to cost in progressive disease. In 7–34 cycles (4.9–23.8 months), the cost of progression-free survival state needed to be changed to cost in maintenance therapy. Since the use of camrelizumab could not exceed 2 years, after 35 cycles (24.5 months), both progression-free survival and progressive disease states in camrelizumab plus chemotherapy group used the input parameter of cost in progressive disease–camrelizumab.

#### 2.7.2. Utility data

The utility values of the health states of progression-free survival and progressive disease in the model were 0.804 and 0.321, respectively, and were derived from a published study on the utility of Chinese NSCLC (16). In addition, the utility values of 0.71 for progression-free survival and 0.67 for progressive disease from published literature were used for scenario analysis (17).

#### 2.7.3. Adverse events

The model included risks of adverse events of Grade 3+, which were reported in >10% of patients in the NCT03134872 trial in either the camrelizumab plus chemotherapy or chemotherapy alone arm (site of injection). The source of the incidence of adverse events was based on the report by Zhou et al. and the management cost data were from published literature and MENET. As neutrophil count decreased and white blood cell count decreased, mecapegfilgrastim could be used at a cost of $455.62 (18). The management cost of Grade 3+ anemia was about $88.76, including whole blood transfusion, blood type detection, and blood matching test. IL-11 could be used for platelet count decrease of Grade 3+; the price of IL-11 was $19.58 per 1.5 mg, and it was continuously used for 14 days (19).

The average cost for each patient to manage adverse events was calculated and incorporated as a one-time cost during the first treatment cycle in the model (20).

### 2.8. Model structure

The Markov model needs to calculate the transition probability between various health states. The calculation process is difficult and cumbersome, and certain assumptions must be made, such as using a fixed natural mortality rate instead of the transition probability to the death state, which, in turn, leads to certain biases in the results (21, 22). The calculation process of the partition survival model is more concise, avoids some unnecessary model assumptions, and is closer to the actual observed data (23). It has been increasingly used in the pharmacoeconomic evaluation of treatment options for advanced or metastatic cancers (24, 25).

**Figure 1:**
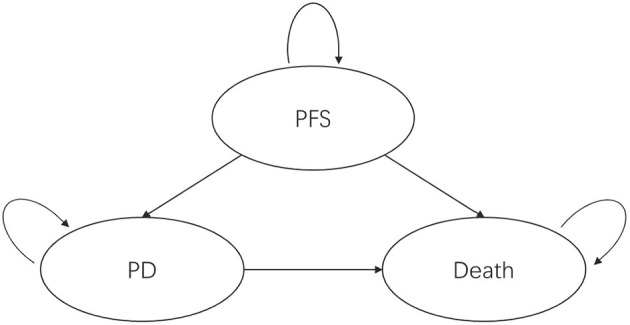
Partitioned survival model health state transitions. PFS, progression-free survival; PD, progressed disease.

A partitioned survival model was established from the perspective of the Chinese healthcare system and divided into three mutually exclusive health states, as shown in Figure 1: *progression-free survival, progressive disease*, and *death* (death). All patients entered the model in the progression-free survival health state; after one cycle, patients could remain in the progression-free survival health state or transition to the progressive disease or dead state, and patients in the progressive disease state could only maintain the progressive disease state or die.

The survival rate during the trial follow-up period was obtained from the survival curve; beyond the follow-up period, the survival rate was extrapolated using the parameter method. The proportion of patients in each healthy state at each time point was calculated as follows:

Progression-free survival: calculated according to the progression-free survival curve.
Progressive disease: proportion of patients who are alive (calculated according to the overall survival curve)—progression-free (calculated according to the progression-free survival curve).
Death: 1—(the proportion of patients who are alive).
The Cycle period of model: 21 days (3 weeks) until 99% of the patients died.

### 2.9. Survival extrapolation

GetData was used to extract points from the progression-free survival and overall survival curves of the two treatment arms. Then, R4.0.4 was used to reconstruct individual patient data using Guyot et al.'s method; exponential, gamma, Weibull, loglogistic, lognormal, and other distributions were used to fit the reconstructed individual patient data (26). The Akaike information criterion (AIC) and Bayesian information criterion (BIC), in combination with visual inspection, were used to select the best-fitting distribution (Table 1). Although the Weibull distribution did not always yield the lowest AIC and BIC statistics within each case, differences in AIC and BIC values were relatively small (< 5% difference), and the Weibull distribution has the advantage of being flexible and variable and has been widely used in tumor survival analysis. Therefore, the Weibull distribution was used to fit the progression-free survival and overall survival curves of camrelizumab plus chemotherapy and chemotherapy alone.

**Table 1:** AIC and BIC values of distributions.

**AIC/BIC**		**Exponential**	**Gamma**	**Gompertz**	**Weibull**	**Loglogistic**	**Lognormal**
Camrelizumab plus chemotherapy group	PFS	748.537/751.860	743.648/750.294	749.050/755.696	744.655/751.301	741.509/748.155	743.952/750.598
	OS	609.002/612.325	595.258/601.904	597.621/604.267	594.876/601.522	595.075/601.721	601.692/608.338
Chemotherapy alone group	PFS	777.973/781.305	774.733/781.399	779.711/786.377	765.382/772.048	770.933/777.599	776.394/783.060
	OS	700.870/704.203	694.045/700.710	697.949/704.615	693.438/700.103	693.169/699.834	693.529/700.195

PFS, progress-free survival; OS, overall survival.

#### 2.9.1. Modeling progression-free survival

**Figure 2:**
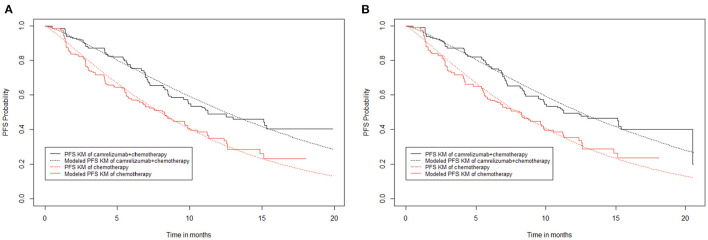
Kaplan–Meier survival curves for progression-free survival. **(A)** Abandon extreme points. **(B)** Keep extreme points.

When curve fitting was performed directly, the progression-free survival of the camrelizumab plus chemotherapy group suddenly dropped at the 20th month, which affected the entire fitting curve moving down (Figure 2B). When attempting to remove this extreme point (Figure 2A), it was found that, compared with the former, it matched better with the original data for 20 months; therefore, curve fitting was performed after the extreme point had been removed. The median progression-free survival periods of camrelizumab plus chemotherapy and chemotherapy alone were 11.30 and 8.30 months, respectively, in the original progression-free survival curve, and 11.27 and 8.25 months, respectively after fitting. The fitting results were consistent with the original results and exhibited high reproducibility. A fitting curve for progression-free survival can be drawn according to the scale and shape parameters above.

In the partitioned survival model, the progression-free survival data of 20.3 months (29 cycles) of the camrelizumab plus chemotherapy group and 18.2 months (26 cycles) of the chemotherapy alone group were obtained directly from the progression-free survival curve, and the follow-up data were calculated from the Weibull distribution.

#### 2.9.2. Modeling overall survival

**Figure 3:**
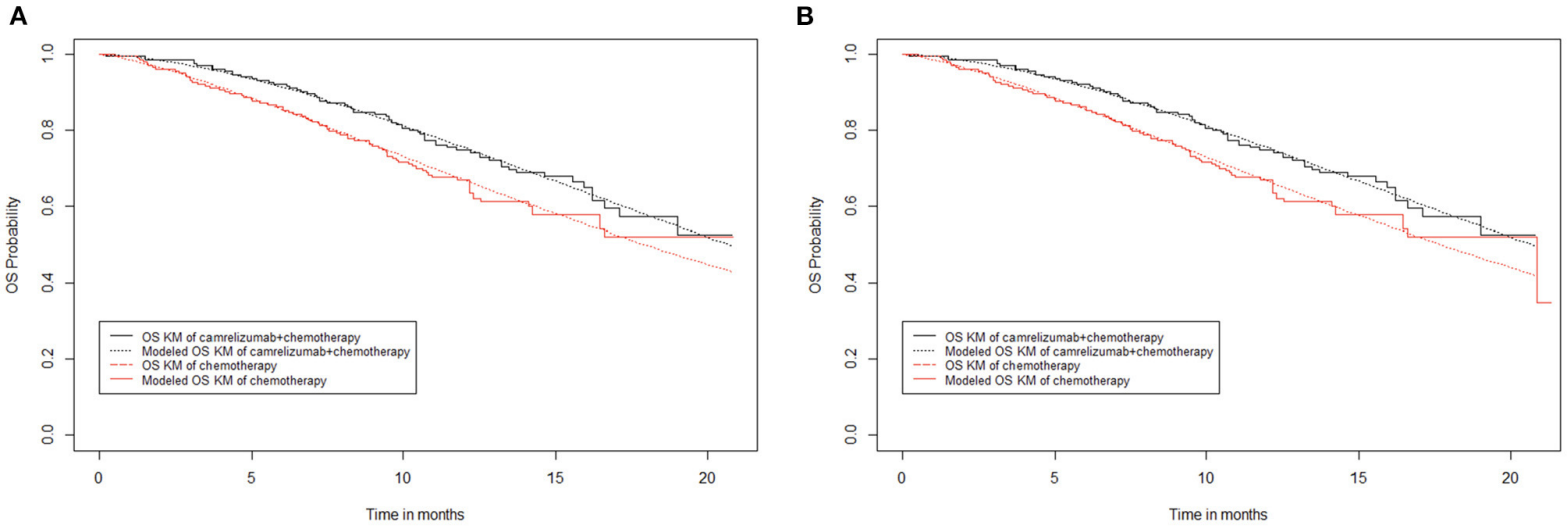
Kaplan–Meier survival curves for overall survival. **(A)** Abandon extreme points. **(B)** Keep extreme points.

The median overall survival of camrelizumab plus chemotherapy in the original overall survival curve was not reached, while that of chemotherapy alone was 20.9 months. However, similar to progression-free survival, there is an extreme value in the chemotherapy alone group of the overall survival curve. When extreme points were retained (Figure 3B), the fitting curve of overall survival in the chemotherapy alone group showed a significant downward shift, and the median survival period of the chemotherapy alone group was 20.9 months. When the extreme points were removed (Figure 3A), the overall survival fitting curve of the chemotherapy alone group was closer to the original data, but the median survival period could not be reached. Comparing the overall survival fitting curves of the above two cases, the difference in survival rate does not exceed 0.01, and it doesn't make much difference to the final result. Thus, in the end, the extreme points were removed in this study.

To be consistent with progression-free survival, the overall survival data of 20.3 months (29 cycles) of the camrelizumab plus chemotherapy group were obtained directly from the overall survival curve, and subsequent data were calculated from the Weibull distribution. The overall survival rate of the chemotherapy alone group remained unchanged after 17 months; to appropriately offset the effect of removing the extreme points, the overall survival data of 18.2 months (26 cycles) in the chemotherapy alone group were obtained directly from the overall survival curve, and subsequent overall survival rate data were calculated from the Weibull distribution.

### 2.10. Subsequent therapies

Subsequent therapy was based on the subsequent 2-line therapy of trial NCT03134872. Subsequent therapies of 3/4/5-line therapy are not considered, because the number of patients in these therapies are fewer than in 2-line therapy. Among the 205 patients in the camrelizumab plus chemotherapy group, 55 patients did not receive subsequent therapy, 75 patients remained on study therapy, and 75 patients received subsequent therapy. Among the 207 patients in the chemotherapy alone group, 52 patients did not receive subsequent therapy, 35 patients remained on study therapy, and 120 patients received subsequent therapy. The subsequent 2-line therapy of trial NCT03134872 include Anti-PD-1 antibody, tyrosine kinase inhibitors, and chemotherapy. The prices of medicines were sourced from MENET (20). Cabozantinib and plinabulin have not yet on the market in China, thus patients use these medicines as subsequent therapies were redistributed. Cabozantinib belong to tyrosine kinase inhibitors therapy, so they were redistributed to afatinib, which is also a tyrosine kinase inhibitors therapy. Plinabulin and docetaxel plus plinabulin belong to chemotherapy, so they were redistributed to docetaxel, which is also chemotherapy. While the costs of subsequent therapies were separately included in the model, overall survival and progression-free survival effects were assumed to be already reflected within the overall survival and progression-free survival Kaplan–Meier data from the NCT03134872 trial.

### 2.11. Analytical methods

#### 2.11.1. Base-case analysis

This study conducted a cost-effectiveness analysis of the two treatment options by constructing a partition survival model, and the model output were total cost, total effectiveness, and incremental cost-effectiveness ratio (ICER). According to the recommendations of the China Guidelines for Pharmacoeconomics Evaluations (2020), three times the GDP per capita of China in 2021 ($35,936.09) was used as the WTP threshold (12). When the ICER is less than the WTP, the higher-cost treatment is more cost-effective than the lower-cost treatment and the increased cost is worth it.

#### 2.11.2. Scenario analysis

This study used the utility value from the literature from Chouaid C et al. who used patient data from Western countries such as Europe and Canada, and the utility values of PFS state and OS state were 0.71 and 0.67, respectively (17). Additionally, Camrelizumab can be reimbursed after entering the Chinese medical insurance list through China's national medical insurance negotiations in 2020, so 20–70% discount is assumed to analyse ICER values in different scenarios.

#### 2.11.3. Sensitivity analysis

To evaluate the effect of the parameter changes, a one-way deterministic sensitivity analysis was conducted over the range of values of the point estimate. The variation range of the parameter in the model was the 95% confidence interval or ±25% of the base value (as shown in Table 2), and the analysis result is presented in the form of a tornado diagram.

**Table 2:** Cost and utility data.

**Drug**	**Dose**	Cost per dose ($)^*^	**Scenario analysis**	**DSA**	**References**
Camrelizumab	200 mg	433.14	20–70% reduction	±25%	(11)
Pemetrexed	500 mg	404.71		±25%	(11)
Carboplatin	50 mg	4.49		±25%	(11)
**Disease management costs**	**Cost**	**Frequency**			**References**
Outpatient fee	3.70	Once every 3 weeks		±25%	#
Blood test	7.40	Once every 3 weeks		±25%	#
Puncture	443.79	Once before treatment			#
PD-L1 test	295.86	Once before treatment			#
Tumor imaging assessments	73.96	Every 6 weeks for the first 54 weeks and every 12 weeks thereafter			#
**Utility**		**Source**	**DSA**	**Scenario analysis**	**References**
PFS	0.804	Beenish Nafees (13)	0.603–0.840	0.71	(14)
OS	0.321	Beenish Nafees (13)	0.050–0.473	0.67	(14)
Discount rate	5%			0–8%	(9)
**Adverse events**	**Cost per patient**	**Incidence of camrelizumab plus chemotherapy group**	**Incidence of chemotherapy group**	**DSA**	**References**
Neutrophil count decreased	455.62	38%	30%	±25%	(5, 11, 15)
White blood cell count decreased	455.62	20%	14%	±25%	(5, 11, 15)
Anemia	88.76	19%	11%	±25%	(5)
Platelet count decreased	5,482.40	17%	12%	±25%	(5, 11, 16)

DSA, deterministic sensitivity analysis; PFS, progress-free survival; PD, progressive disease; OS, overall survival.

^*^All prices are calculated at the exchange rate of USD 1 per RMB 6.76.

^#^Data were collected from local hospitals.

To assess the robustness of the model results, probabilistic sensitivity analysis was performed to set a specific distribution for each parameter (the cost is a gamma distribution and the utility is the beta distribution) (Table 3). After 1,000 Monte Carlo simulations, scatter plots and cost-effectiveness acceptability curves are presented.

**Table 3:** Sensitivity analysis parameters.

	**Base-case value**	**Probabilistic sensitivity analysis**
One-time cost–camrelizumab	1,952.78	GAMMA distribution with the SE set at 20% of the base-case value
Cost in PFS–camrelizumab	904.48	GAMMA distribution with the SE set at 20% of the base-case value
Cost in maintenance therapy–camrelizumab	837.13	GAMMA distribution with the SE set at 20% of the base-case value
Cost in PD–camrelizumab	388.96	GAMMA distribution with the SE set at 20% of the base-case value
One-time cost–chemotherapy	1,607.77	GAMMA distribution with the SE set at 20% of the base-case value
Cost in PFS–chemotherapy	774.54	GAMMA distribution with the SE set at 20% of the base-case value
Cost in maintenance therapy–chemotherapy	707.19	GAMMA distribution with the SE set at 20% of the base-case value
Cost in PD–chemotherapy	421.67	GAMMA distribution with the SE set at 20% of the base-case value
PFS	0.804	BETA distribution with the SE set at 20% of the base-case value
PD	0.321	BETA distribution with the SE set at 20% of the base-case value

PFS, progress-free survival; PD, progressive disease; ICER, incremental cost-effectiveness ratio; SE, standard errors.

## 3. Results

### 3.1. Base-case analysis

According to the results of the base-case analysis in Table 4, camrelizumab plus chemotherapy provided 1.98 QALYs at a cost of $28,289.85, whereas chemotherapy alone provided 1.57 QALYs at a cost of $17,807.74. Compared with chemotherapy alone, the incremental discounted costs of camrelizumab plus chemotherapy were $10,482.12, and the discounted QALYs gained were 0.41. As calculated, the ICER comparing the camrelizumab plus chemotherapy-arm to the chemotherapy alone-arm was $25,375.96 per QALY, well below than the three-times GDP per capita of China in 2021 ($35,936.09), which is the threshold used to define cost-effectiveness between the two arms.

**Table 4:** Cost-effectiveness of treatment of camrelizumab + chemotherapy vs. chemotherapy in full trial population.

	**Chemotherapy**	**Camrelizumab + chemotherapy**	**Incremental camrelizumab + chemotherapy vs. chemotherapy**
Costs	17,807.74	28,289.85	10,482.12
QALYs	1.57	1.98	0.41
**ICER**			**25,375.96**

QALY, quality-adjusted life year; ICER, incremental cost-effectiveness ratio.

### 3.2. Scenario analysis

In addition to the utility value of the Chinese population, we also used the utility value from Chouaid et al. (17). They used patient data from Western countries such as Europe and Canada. This value was usually used to measure the cost-effectiveness analysis of NSCLC before there is a utility value for Chinese people (27, 28). The results show that incremental discounted QALY changed from 0.41 to 0.18, and ICER changed to $58,808.14 per QALY, which is higher than the three-times GDP per capita of China in 2021 ($35,936.09).

Camrelizumab enters the Chinese medical insurance list through China's national medical insurance negotiations in 2020, which means that patients with NSCLC can be reimbursed at a price of $433.14. Therefore, a hypothetical reduction in the price of camrelizumab by 20–70% resulted in an ICER of $21,382.83/QALY to $11,400.01/QALY, far less than the GDP per capita of China in 2021 ($35,936.09) (Table 5).

**Table 5:** Cost-effectiveness of treatment of camrelizumab + chemotherapy vs. chemotherapy in scenario analysis.

	**Incremental costs**	**Incremental QALYs**	**ICER**
**PFS: 0.71; PD: 0.67**			
	10,482.12	0.18	58,808.14
**Simulation of reduction of camrelizumab selling price**			
20% reduction	8,832.66	0.41	21,382.83
30% reduction	8,007.94	0.41	19,386.27
40% reduction	7,183.21	0.41	17,389.70
50% reduction	6,358.48	0.41	15,393.14
60% reduction	5,533.76	0.41	13,396.57
70% reduction	4,709.03	0.41	11,400.01

PFS, progress-free survival; QALY, quality-adjusted life year; ICER, incremental cost-effectiveness ratio.

### 3.3. Sensitivity analysis

#### 3.3.1. Deterministic sensitivity analyses

**Figure 4:**
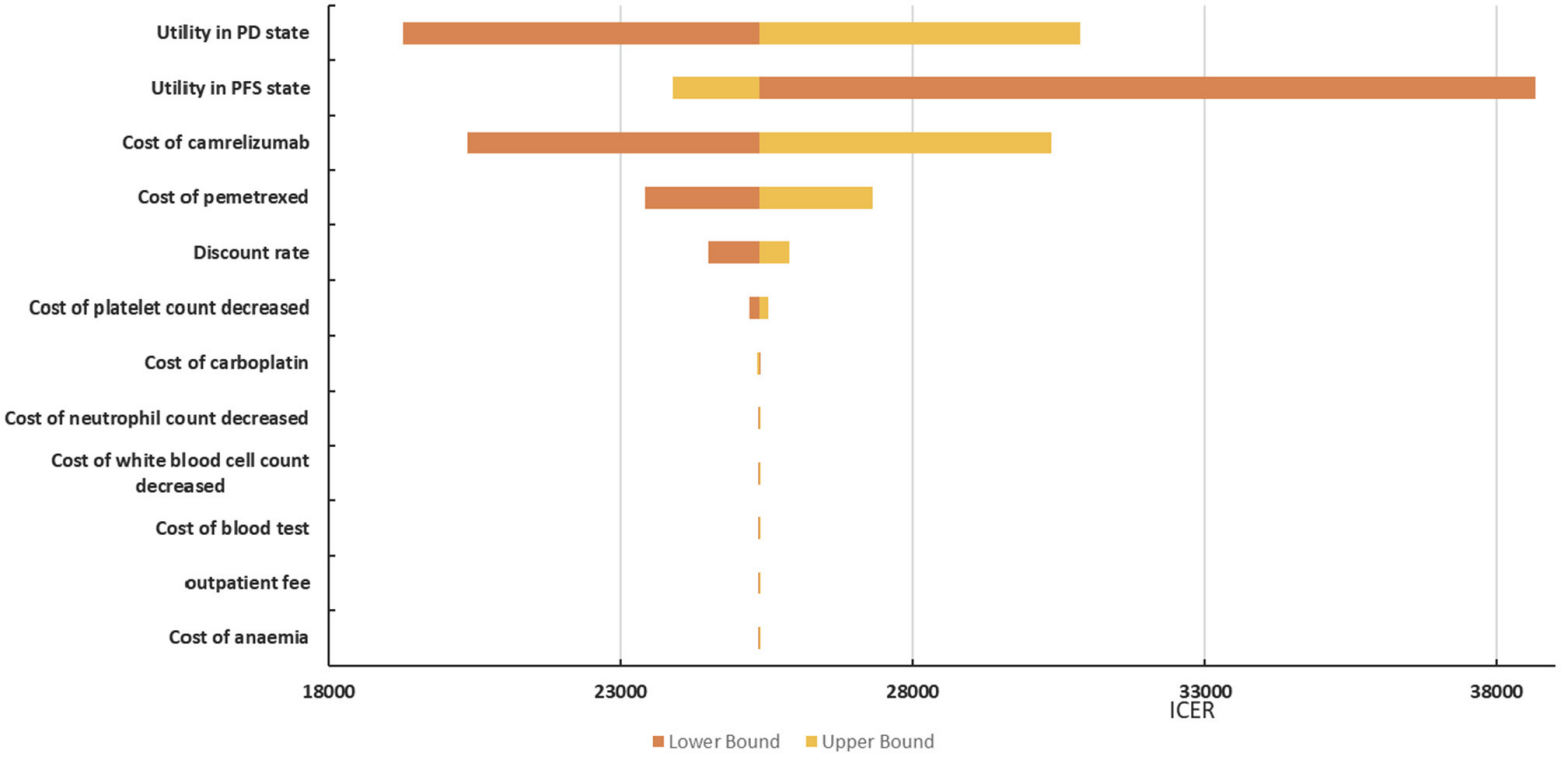
Tornado diagram for ICER of camrelizumab + chemotherapy vs. chemotherapy.

The tornado diagram depicted in Figure 4 shows the effects of individual parameters on the ICER. The parameters that have the greatest influence on the ICER were the utility values of the progression-free survival state, the utility values of the progressive disease state, and the price of camrelizumab. In addition, management costs for adverse events, such as anemia, decreased white blood cell count, decreased neutrophil count, and decreased platelet count, had a weaker effect on the ICER (difference < 5%). In general, the ICER ranged between $25371.66/QALY and $38668.46/QALY. In addition to the ICER is $38668.46/QALY when the utility value of progression-free survival state is 0.603, which exceeds the willingness to pay threshold. The others are less than the three-times GDP per capita of China in 2021 ($35,936.09).

#### 3.3.2. Probabilistic sensitivity analysis

**Figure 5:**
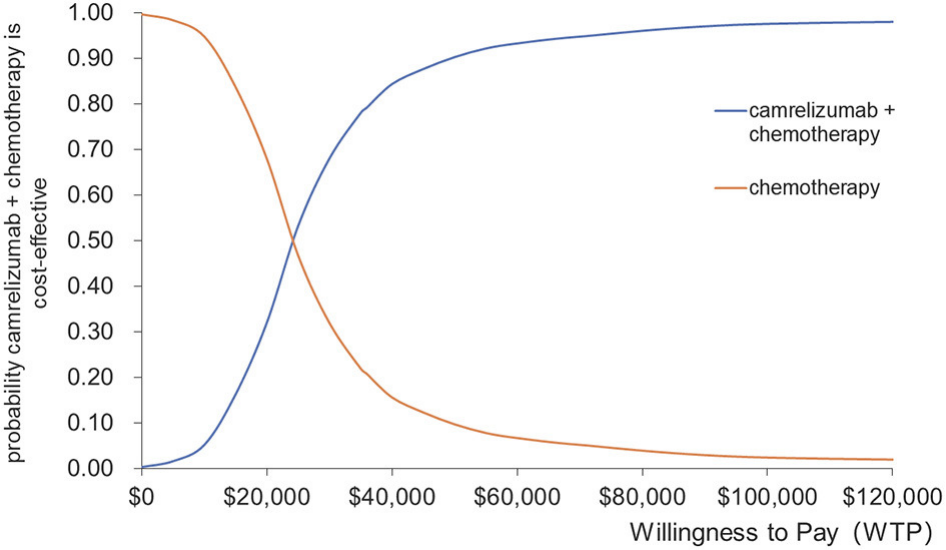
Cost-effectiveness acceptability curve for camrelizumab + chemotherapy vs. chemotherapy.

**Figure 6:**
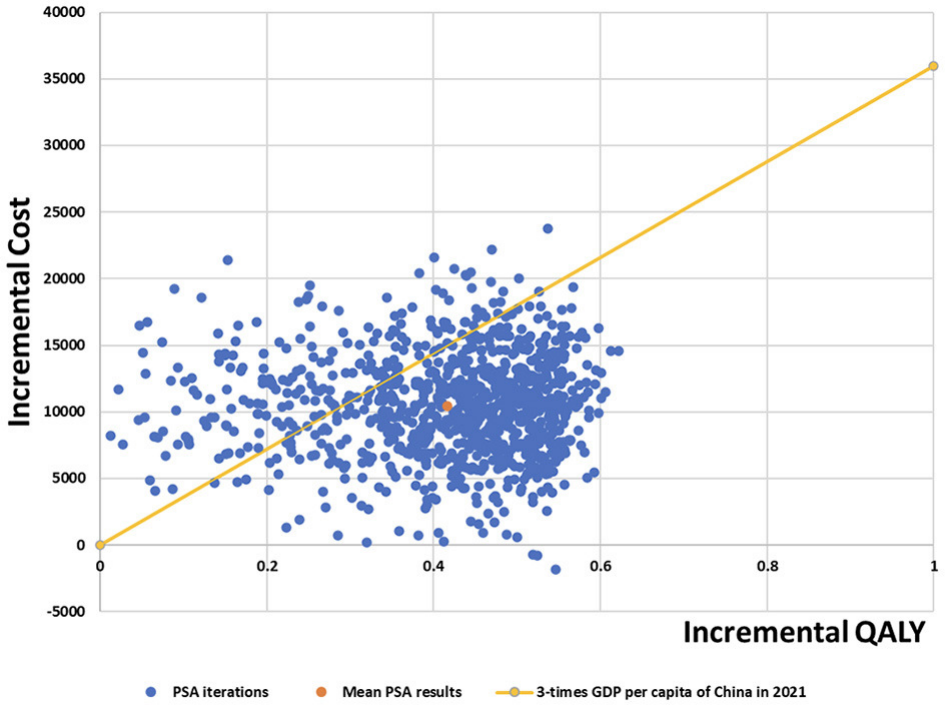
Probabilistic sensitivity analysis scatter plot.

The results of the probabilistic sensitivity analysis are presented in the form of a cost-effectiveness acceptability curve and scatter plots, as shown in Figures 5, 6. Camrelizumab plus chemotherapy became more economical with an increase in the willingness-to-pay threshold. At the willingness-to-pay threshold of $24,000/QALY, two curves intercrossed, which meant that camrelizumab plus chemotherapy had a 50% chance of being cost-effective compared with chemotherapy alone. When the willingness-to-pay threshold reaches $35,936.10/QALY (three times the GDP per capita of China in 2021), the probability of cost-effectiveness of camrelizumab plus chemotherapy was approximately 80%.

The mean incremental cost of probabilistic sensitivity analysis was $10,431.58; mean incremental QALYs was 0.42; and mean expected ICER was $25,047.68/QALY for camrelizumab plus chemotherapy compared with chemotherapy alone. Scatter plots of probabilistic sensitivity analysis showed that most of the cost-effectiveness points fell below the three-times GDP per capita of China in 2021. Therefore, the results of the probabilistic sensitivity analysis were consistent with those of the base-case analysis, indicating that the results of the base-case analysis were robust.

## 4. Discussion

The present analysis estimated the cost-effectiveness of camrelizumab plus chemotherapy vs. chemotherapy alone in first-line treatment of metastatic or advanced non-squamous NSCLC without targetable EGFR or ALK genetic aberrations in China based on data from NCT03134872. The base-case analysis indicated that incremental costs and incremental QALYs were $10,482.12 and 0.41. The reported ICER was $25,375.96/QALY, which is much lower than China's per capita GDP in 2021. The ICER for sintilimab plus chemotherapy was $31,556/QALY (29). The first-line tislelizumab plus pemetrexed-platinum (TPP) was $28,749/QALY (30). According to the *China Guidelines for Pharmacoeconomics Evaluations (2020)*, the willingness-to-pay threshold of China is estimated to be approximately estimated as the three-times GDP per capita of China in 2021 ($35,936.09). The ICER of camrelizumab plus chemotherapy fell within these ranges of acceptable thresholds in both the base-case analysis and scenario analysis. The final ICER value is quite different in previous literatures. Firstly, it is because of the choice of utility value. Different patient groups have their preferences for various health states, and it can be confirmed from the results of this paper that the variation of utility data has a great impact on ICER. The second reason is the choice of subsequent therapies. There are differences in the design of subsequent therapies in previous literatures, and use some expensive immunotherapy drugs such as nivolumab as subsequent therapies, which will also have a great impact on ICER value.

The main driver of the increased cost of camrelizumab plus chemotherapy was additional medical costs. The results of deterministic sensitivity analysis illustrated that the ICER was most sensitive to utility values, the price of camrelizumab and pemetrexed, and the discount rate. In general, the ICER ranged between $25371.66/QALY and $38668.46/QALY in deterministic sensitivity analysis. Only the lower progression-free survival utility value resulted in ICER values higher than the threshold, and the others were within the acceptable threshold range. Results from the probabilistic sensitivity analysis illustrated 80% probability that the ICER would be below $35,936.09/QALY; the mean expected ICER of probabilistic sensitivity analysis was consistent with base-case analysis, confirming the credibility of the results. Therefore, camrelizumab plus chemotherapy is more cost-effective than chemotherapy.

This research was aimed at the Chinese people; hence, in the base-case analysis, the utility value of Chinese was selected, and the utility value from Western countries was used as a scenario analysis, as it had been quoted many times and is more authoritative (31, 32). Camrelizumab had an incremental QALY of 0.41 compared with chemotherapy alone when the utility value came from Chinese. The incremental QALY was 0.18 when the utility value came from Western countries. The result of scenario analysis illustrates that the ICER is higher than WTP which is some different from base-case analysis results. The deterministic sensitivity analysis shows that the parameters that have the greatest influence on the ICER were the utility values of the progression-free survival state, the utility values of the progressive disease state, the price of camrelizumab, and the price of pemetrexed. The decrease the utility values of PD state, cost of camrelizumab and pemetrexed will reduce the ICER, while the decrease of the utility values of PFS state will increase the ICER value. Utility value refers to patients' preference for the health states brought by a certain intervention measure, which has a great influence on the result of ICER. Camrelizumab and pemetrexed are relatively expensive drugs that continue to be used in the course of patient treatment, and changes in their prices will also cause large fluctuations in ICER value.

For the cost-effectiveness analysis of camrelizumab plus chemotherapy, all data, such as clinical survival, cost, and utility value data, came from China in order to reflect the region best. We hope that future research on Chinese populations will expand and diversify the data available for further healthcare studies.

Nevertheless, our study has three limitations as well. First, the current model assumes that camrelizumab cannot be used for >2 years (5). However, when camrelizumab is used for >2 years, the treatment cost will increase, but the survival benefit will not increase in the same proportion with the cost (25), so its ICER value will increase slightly.

Secondly, in subsequent therapies of the chemotherapy alone group, 86 patients (41.5%) used PD-1 inhibitors for continued treatment (such as camrelizumab, nivolumab, etc.) (5). Considering the high proportion of these therapies, so it was proportionally included in the model in this study. The percentage of patients receiving any other specific therapies was also included according to the proportion in the trial NCT03134872. As these subsequent treatments occurred outside the clinical trial and which made a hardly impact on the results of this study (20). Thus, the duration and cost of these subsequent therapies were included without adjusting the Kaplan–Meier curve in this study for assuming the OS and PFS impacts were to be already reflected within the OS and PFS Kaplan–Meier curves from NCT03134872.

Thirdly, camrelizumab has not been on the market for a long time, so the median overall survival of the camrelizumab plus chemotherapy group did not reach in trial NCT03134872, which would lead to less rigorous results. In the trial NCT03134872, the clinical trial time of the camrelizumab plus chemotherapy group has been 20 months, while the median overall survival is approximately 21 months in the model of this study. Anyway, when the follow-up time of the clinical trial is extended, the median overall survival time of the camrelizumab plus chemotherapy group can be reached, and the result of curve fitting will be more accurate and credible.

## 5. Conclusions

We reported the cost-effectiveness of camrelizumab plus chemotherapy compared with chemotherapy alone in first-line treatment of patients with metastatic or advanced non-squamous NSCLC without targetable EGFR or ALK genetic aberrations in China. From the Chinese healthcare perspective, the base-case analysis illustrated that the ICER of camrelizumab plus chemotherapy was $25,375.96/QALY. Based on these results and the three-times GDP per capita of China in 2021 ($35,936.09), camrelizumab could be considered as a cost-effective option compared with chemotherapy. These findings provide a reference for local clinical decision-making and for Chinese medical insurance negotiations in the future.

## Data availability statement

The original contributions presented in the study are included in the article/supplementary material, further inquiries can be directed to the corresponding author.

## Author contributions

HD, WW, and YC developed the economic model and performed the analyses. HD, WW, and XF interpreted the results and wrote the draft manuscript. HD, WW, XF, and YC reviewed, analyzed, and interpreted the data. HD and YC contributed to the design of the primary model and the interpretation of the results. All authors reviewed and approved the final version.
